# The Power of the Web in Cancer Drug Discovery and Clinical Trial Design: Research without a Laboratory?

**DOI:** 10.4137/cin.s3191

**Published:** 2010-02-18

**Authors:** Christine Galustian, Angus G. Dalgleish

**Affiliations:** Department of Oncology, Division of Cellular and Molecular Medicine, St. Georges University of London, Cranmer Terrace, London SW17 0RE. Email: cgalusti@sgul.ac.uk

**Keywords:** cancer, drug discovery, data mining, clinical trials

## Abstract

The discovery of effective cancer treatments is a key goal for pharmaceutical companies. However, the current costs of bringing a cancer drug to the market in the USA is now estimated at $1 billion per FDA approved drug, with many months of research at the bench and costly clinical trials. A growing number of papers highlight the use of data mining tools to determine associations between drugs, genes or protein targets, and possible mechanism of actions or therapeutic efficacy which could be harnessed to provide information that can refine or direct new clinical cancer studies and lower costs. This report reviews the paper by R.J. Epstein, which illustrates the potential of text mining using Boolean parameters in cancer drug discovery, and other studies which use alternative data mining approaches to aid cancer research.

## Commentary

The discovery of effective treatments for cancer represents a key goal for pharmaceutical companies who wish to identify drugs that can prolong survival time and even reverse cancers, while having an acceptable toxicity profile. However, the average cost of bringing a drug to the commercial market in the USA is now estimated at $1 billion per FDA approved drug, and many factors have compounded the expense of these developments such that cancer drug discovery is now both extremely slow and costly even for a potential blockbuster. Among the many factors contributing to the cost are the high price of clinical trial organisation and the bench research hours required to validate the efficacies and toxicities associated with a drug despite the use of time saving technologies such as high throughput screening to determine efficacies and genomic analyses of drug effects. A growing number of papers highlight the use of data mining tools to determine associations between drugs, genes or protein targets, and possible mechanism of actions or therapeutic efficacy which could be harnessed to provide information that can refine or direct new clinical cancer studies.

One common method of data mining is referred to as text mining. Richard Epstein^1^ provides a number of examples of how text mining using Boolean terms can be used to determine associations between a cancer type or drug and the symptoms or efficacies observed. For example he describes how phenotypes and environmental factors associated with either squamous cell carcinoma or adenocarcinoma (e.g. smoking and lymph node metastasis for squamous cell carcinoma vs. hormone and liver metastasis for adenocarcinoma) can be deciphered. Mechanistic associations of different drugs such as tyrosine kinase inhibitors and metalloprotease inhibitors can also be calculated: Growth or replication is more strongly associated with tyrosine kinase inhibitors and invasion and metastasis inhibition is more strongly associated with metalloprotease inhibitors.

Epstein also provides examples of how text mining can determine associations between types of cancers and a particular gene for example, AKT.^2^ The gene for AKT encodes a retroviral protein which is a pivotal cell signalling protein which when activated leads to inhibition of cellular apoptosis and activation of its downstream target (mammalian target of rapamycin (mTOR)), which increases mRNA translation through combination with its protein RAPTOR (regulatory-associated protein of mTOR). By text mining associations, AKT is associated with a number of cancers of which the most prominent is prostate cancer. When the association is then compared with cancers in which the mTOR inhibitor temsirolimus has been used, prostate cancer also gives the strongest correlation as the disease in which this drug has been most commonly used. Therefore, text mining can detect correlations between specific cancers and their associated gene defects and the drugs that are used for that cancer. A number of papers have shown how text mining has contributed greatly to identifying critical genes and drugs in a number of cancers. For example Pospisil et al have used a combined textual-structural mining approach to identify potential enzyme targets in the extracellular space of cancerous cells for six common, lethal human tumors, by searching databases such as PubMed abstracts, NCBI Entrez, UniProt, (a universal gene/protein database) and Interpro, a conserved protein domains database. By using keyword and gene ontology terms and by clustering these terms to specific cell locations, a list of cancer-related hydrolases for each tumor type have been identified as therapeutic targets including prostatic acid phosphatase (ACPP also known as PAP), prostate-specific antigen (PSA) and sulfatase 1 (SULF1).^3^,^4^ Another study by Turk et al have used text mining of National Cancer Institute’s DTP drug repository to search for compounds showing increased toxicity in MDR cells and discovered 22 compounds with MDR specific toxicity, and a further 15 drugs showing increased cytotoxicity in cells with P-glycoprotein. Analysis of these compounds has led to the formulation of structure activity relationships linking mechanism of action with metal chelation, and shows that p-glycoprotein is not the only target of compounds that are effective in MDR cells.^5^

However, there are a few known limitations to text mining. Associations are based on the use of constant terminology for a drug or gene, whereas this may not be the case for example, where drug names are changed by companies. Also there is restricted access to full text journals and also restriction to abstract publications and some chemical and physical science journals in databases such as pubmed so that data associations need to be retrieved from a more limited source of overall citations. Text mining may therefore be better utilized by combining it with other data mining tools such as microarray database mining. This uses microarray gene data from experiments which have analysed genomes or sets of genes of particular cells or tissues.^6^ This allows the discovery of drug sensitive and disease specific genes which can be used to identify targets for cancer therapy. Such analyses yield vast amounts of gene data as even a whole genome can be available on a chip. When text mining and microarray data mining are combined, powerful analyses of data can be applied to decipher cancer therapy targets. For example, Ho and colleagues have identified a set of 64 genes that are specifically expressed in endothelial cells compared with non endothelial cell types from combined text mining and microarray analyses.^7^ There are also caveats that can be applied to microarray mining.^8^ Microarray analysis results in a vast amount of gene data from a dataset of samples that is normally at least 100 fold less than the gene data generated. There is also the problem of noise where artifactual expression may be seen with platforms that are not stringent enough to filter outlier data, and the requirement for repeat array analysis which sometimes shows variance in the fold change produced in single genes.^9^ This type of analysis always requires a post test to confirm the gene changes observed, either quantitative pcr or western blotting of the associated proteins. Microarray gene data can be complemented by proteomic data analysis such as mass spectrometric analysis, SELDI-TOF (Surface-enhanced laser desorption/ionization-time of flight) and bio-plex technologies which allow analysis of very large numbers of proteins on an array format and combined analyses of proteins and genes (often referred to as pathway analyses). These techniques both complement and strengthen the observations seen with the genes alone. For example, this approach has been used to determine biomarkers which can give a very early prognosis of ovarian cancer.^10^ Such an approach has also recently helped to lead to discovery of biomarkers which can predict a favourable response to prostate cancer vaccines (Bodman-Smith et al, paper in preparation).

The power of data mining has now been harnessed by a growing industry specialising in the production of databases which can utilise text or gene or protein data. A selection of databases with direct application to cancer drug or target molecule discovery are presented in Table 1.

The tools that these databases provide for both the drug industry and academia can maximise the mining process compared to manual mining techniques. Whereas data mining has not yet resulted in blockbuster discovery on its own merit, the use of this technology harnessed with the power of dedicated databases and bench top research, has already contributed to deciphering mechanisms of action of genes and drugs and should allow a much more rapid progress toward discovery of effective cancer therapies in the future.

## Figures and Tables

**Table 1.** A selection of databases with direct application to cancer drug and target molecule discovery.

**Database/resource used**	**Mining tool used**	**Reference/website**	**Notes**
Clinical trials.gov	Text	http://clinicaltrials.gov/	A list of clinical trials taking or taken place: currently 80,055 trials with locations in 170 countries
Pubmed	Text	http://www.ncbi.nlm.nih.gov/pubmed	Public access but no abstract publications available on this database
Web of knowledge	Text	http://wok.mimas.ac.uk/	Abstract publications + full papers available
Oncomine	Gene array data	https://www.oncomine.org/resource/login.html	Largest cancer specific microarray based database. (48,000,000 gene expression measurements from over 4700 microarray experiments
CGAP database	Gene array data	www.ncbi.nlm.nih.gov/ncicgap.	Contains cancer specific cDNA libraries, clones, and sequence data in addition to microarray data. Public access
Gene expression atlas	Gene array data	http://www.geneatlas.org	Uses gene expression data from tissue samples
Genemap	Gene array data	http://robotics.stanford.edu/~erans/cancer/	Run by Stanford university: 1975 published microarrays spanning 22 tumor types
Pathway studio	Gene array data and text	http://www.ariadnegenomics.com/products/pathway-studio/	Private software program integrating gene array and text mining analyses to identify pathways of action for genes/drugs
Open proteomics database	Mass spectrometry data	http://bioinformatics.icmb.utexas.edu/OPD/	Public database for storing and disseminating mass spectrometry based proteomics data. The database currently contains roughly 3,000,000 spectra representing experiments from 5 different organisms
EMBL proteomic database (Pride database)	Mass spectrometry data	http://www.ebi.ac.uk/pride/	PRIDE currently contains: 9,964 experiments, 2,564,320 proteins, 12,015,539 peptides 1,753,906 unique Peptides 53,348,019 Spectra
NCI clinical proteomics program	Seldi-Tof data	http://home.ccr.cancer.gov/ncifdaproteomics/ppatterns.asp	Restricted at present to seldi-tof data sets from ovarian and pancreatic cancer data sets. Just data sets and not a database as such at present
